# Urinary biomarkers in the early diagnosis of diabetic kidney disease and diabetic retinopathy: A pilot study

**DOI:** 10.5937/jomb0-61991

**Published:** 2026-05-22

**Authors:** Brankica Terzić, Zoran Radojičić, Predrag Đurić, Dušan Pualić, Mirko Resan, Mirjana Mijušković

**Affiliations:** 1 University of Defense Belgrade, School of Medicine, Military Medical Academy, Clinic of Nephrology, Belgrade; 2 University of Belgrade, Faculty of Organizational Sciences, Laboratory for Statistics, Belgrade; 3 University of Defense Belgrade, School of Medicine, Clinic of Cardiology, Belgrade; 4 Military Medical Academy, Clinic of Nephrology, Belgrade; 5 University of Defense Belgrade, School of Medicine, Military Medical Academy, Clinic of Ophthalmology, Belgrade

**Keywords:** diabetes mellitus, microalbuminuria, biomarkers, type IV collagen, transferrin, L-FABP, dijabetes melitus, mikroalbuminurija, biomarkeri, kolagen tip IV, transferin, L-FABP

## Abstract

**Background:**

Diabetic kidney disease (DKD) and diabetic retinopathy (DR) are the most frequent microvascular complications of diabetes mellitus. Although microalbuminuria is a conventional early marker of DKD, its limited sensitivity and the growing recognition of nonalbuminuric DKD highlight the need for more sensitive early detection tools. Urinary biomarkers such as type IV collagen, transferrin, and liver-type fatty acid-binding protein (L-FABP) may capture early glomerular or tubular injury before the onset of overt albuminuria. This pilot study evaluated the diagnostic performance of these biomarkers in adults with type 2 diabetes mellitus (T2DM) and explored their associations with DR.

**Methods:**

A cross-sectional per-protocol study included 80 T2DM patients and 10 healthy controls. Diabetic participants were categorised as normoalbuminuric (&lt;30 mg/day) or microalbuminuric (30-300 mg/day). First-morning urine samples were used for final analyses. Urinary type IV collagen, transferrin, and L-FABP were quantified using ELISA and expressed in ng/mL. Group comparisons used t-tests or ANOVA for normally distributed variables, and Mann-Whitney U or Kruskal-Wallis tests for non-normally distributed data. Correlations were assessed using Pearson or Spearman coefficients. Diagnostic performance was analyzed using ROC curves.

**Results:**

The cohort (55% male; mean age 59.85± 8.87 years) had similar BMI, smoking status, and eGFR across groups. Biomarker levels differed significantly among microalbuminuric, normoalbuminuric, and control groups (p&lt;0.001): type IV collagen (0.97 vs. 0.55 vs. 0.48), transferrin (45.51 vs. 14.83 vs. 6.57), and L-FABP (480.15 vs. 228.08 vs. 89.91). Type IV collagen demonstrated the best diagnostic ability for early DKD (AUC 90.4%). DR was present in 24 patients (30%), and transferrin levels were significantly higher in those with DR (81.38 vs. 38.12 ng/mL; p=0.03).

**Conclusions:**

Urinary type IV collagen, transferrin, and L-FABP show promise for early DKD detection, while transferrin additionally correlates with DR. Larger studies are needed to confirm these findings.

## Abstract (sr)

**Uvod:**

Dijabetesna bolest bubrega (DBB) i dijabetesna retinopatija (DR) su najčešće mikrovaskularne komplikacije dijabetesa melitusa. Mikroalbuminurija je standardni marker ranog oštećenja bubrega, ali njena ograničena osetljivost, naročito uz sve veću učestalost nealbuminurične DBB, naglašava potrebu za pouzdanijim biomarkerima. Urinarni kolagen tipa IV, transferin i L-FABP mogu da ukažu na ranu glomerularnu ili tubularnu leziju pre pojave albuminurije. Cilj ove pilot-studije je bio da proceni dijagnostičku vrednost navedenih biomarkera kod osoba sa dijabetesom tipa 2 (T2DM) i ispita njihovu povezanost sa DR.

**Metode:**

U per-protokol, pilot studiju preseka uključeno je 80 odraslih osoba sa T2DM i 10 zdravih kontrola. Ispitanici su svrstani u normoalbuminuričnu (&lt;30 mg/dan) i mikroalbuminuričnu (30-300 mg/dan) grupu. Prikupljeni su uzorci prvog jutarnjeg i 24-časovnog urina, ali su analize sprovedene na prvim jutarnjim uzorcima zbog odsustva značajnih razlika. Koncentracije kolagena tipa IV, transferina i L-FABP su određene ELISA metodom. Statistička obrada je obuhvatila testove normalnosti raspodele, parametarske i neparametarske testove, korelacione analize i ROC krive.

**Rezultati:**

Prosečna starost pacijenata bila je 59,85± 8,87 godina, bez razlika u BMI-u, pušačkom statusu i eGFR-u među grupama. Sva tri biomarkera su bila značajno viša u mikroalbuminuričnoj u odnosu na normoalbuminuričnu i kontrolnu grupu (p&lt; 0,001): kolagen tipa IV (0,97 vs. 0,55 vs. 0,48 ng/mL), transferin (45,51 vs. 14,83 vs. 6,57 ng/mL) i L-FABP (480,15 vs. 228,08 vs. 89,91 ng/mL). Kolagen tipa IV je pokazao najveću dijagnostičku tačnost (AUC 90,4%). DR je registrovana kod 24 pacijenta (30%), a transferin je bio značajno viši kod obolelih (81,38 vs. 38,12 ng/mL; p=0,03).

**Zaključak:**

Urinarni kolagen tipa IV, transferin i L-FABP mogu da doprinesu ranom otkrivanju DBB, dok transferin pokazuje dodatnu povezanost sa DR. Potrebne su veće studije radi potvrde ovih nalaza.

## Introduction

Diabetes mellitus (DM) is a chronic disease with a profound global impact, affecting approximately 589 million people, a number expected to rise to 853 million by 2050, representing a global prevalence of 10% [1]. Among its complications, diabetic kidney disease (DKD) and diabetic retinopathy (DR) are the most common microvascular complications.

DR is a microvascular complication of DM that damages retinal capillary endothelial cells, leading to increased vascular permeability, ischemia, and neovascularization. In clinical practice, it is usually divided into proliferative diabetic retinopathy and non-proliferative diabetic retinopathy according to the presence of retinal neovascularization [2]. DR is the leading cause of vision loss in developed countries [3]. If current trends continue, the number of individuals with vision-threatening DR is projected to increase from 37.3 million to 56.3 million by 2045 [4][5]. The kidney has similarities to the eye in terms of origin, development, and structure. Both the glomeruli and choroid have extensive vascular networks of similar structures; the inner retina and the glomerular filtration barrier share similar developmental pathways, and renin-angiotensin-aldosterone cascades have been found in both the kidney and retina [6].

DKD, the most prevalent microvascular complication of DM, is the leading cause of chronic kidney disease [7]. It is characterised by increased urinary albumin excretion, elevated serum creatinine, and reduced glomerular filtration rate [8]. The pathogenesis of DKD involves complex interactions among glomerular, tubular, interstitial, and vascular compartments, primarily mediated by hyperglycemia-induced oxidative stress, inflammation, and dysregulation of growth factors [9][10]. Early pathological features include glomerular hyperfiltration, glomerular basement membrane thickening, mesangial expansion, and progressive albuminuria [11]. Microalbuminuria is widely regarded as the first clinical marker of DKD. It may persist for years and frequently progresses to overt proteinuria, which accelerates renal function decline [12]. Screening is typically performed using the urinary albumin-to-creatinine ratio, with thresholds of ≤30 mg/g (normal), 30-300 mg/g (microalbuminuria), and >300 mg/g (macroalbuminuria) [13]. However, a subset of patients develop DKD without elevated urinary albumin levels, underscoring the need for more sensitive and specific biomarkers [14].

Several glomerular and tubular biomarkers have been investigated as potential early indicators of DKD, including transferrin, type IV collagen, and liver-type fatty acid-binding protein (L-FABP) [15][16]. The biological plausibility of these biomarkers is supported by experimental and clinical data: type IV collagen reflects glomerular basement membrane injury [17][18]. Transferrin, a negatively charged protein, is filtered more readily in early glomerular damage, and it may correlate with DR [19][20][21]. L-FABP mirrors proximal tubular stress and urinary concentrations reflecting the severity of DKD [22][23][24]. However, translating renal biomarkers to retinal pathology is complex, and the relationships must be interpreted with caution.

Given the unmet need for improved early detection strategies, this pilot study assessed the diagnostic value of urinary type IV collagen, transferrin, and L-FABP in patients with type 2 diabetes and explored their associations with DR.

## Materials and methods

### Study design and population

This per-protocol cross-sectional pilot study included 80 adults with type 2 diabetes mellitus (T2DM) and 10 healthy controls. All diabetic patients had a disease duration of at least one year and an estimated glomerular filtration rate (eGFR) >60 mL/min/1.73 m^2^. Participants were categorised into normoalbuminuric (≤30 mg/day) and microalbuminuric (30-300 mg/day) groups.

### Clinical assessment

Patients underwent standardised clinical evaluation, including anthropometrics, blood pressure measurements, renal ultrasonography, and ophthalmologic examination. Laboratory testing included fasting glucose, urea, creatinine, lipid profile, and HbA1c.

### Urine sampling and biomarker measurement

Both 24-hour urine and first-morning urine samples were collected initially. Since no statistical differences were observed between first-morning and 24-hour samples, only first-morning samples were used for final analyses. Urinary type IV collagen, transferrin, and L-FABP were measured using ELISA. Values were expressed as absolute concentrations (ng/mL).

### Statistical analysis

Normality was assessed using the Kolmogorov-Smirnov test. Continuous variables were compared using Student's t-test or ANOVA for normally distributed variables, and Mann-Whitney U or Kruskal-Wallis tests for non-normally distributed data. Pearson or Spearman correlation coefficients were applied where appropriate. Diagnostic performance was assessed using ROC curves. A post-hoc power analysis demonstrated >85% power to detect group differences in the primary biomarkers.

### Statistics

All statistical analyses were conducted using SPSS software, version 23.0. Normality was assessed using the Kolmogorov-Smirnov test. Continuous variables were compared using Student's t-test or ANOVA for normally distributed variables, and Mann-Whitney U or Kruskal-Wallis tests for non-normally distributed data. Pearson or Spearman correlation coefficients were applied where appropriate. Diagnostic performance was assessed using ROC curves. A post-hoc power analysis demonstrated >85% power to detect group differences in the primary biomarkers.

## Results

A total of 80 patients were enrolled in the study, including 44 males (55%) and 36 females (45%), with a mean age of 59.85±8.87 years (range 38-73). The mean duration of diabetes was 13.29±7.69 years, and the average estimated glomerular filtration rate (eGFR) was 86.86±14.18 ml/min/1.73 m^2^.

Patients were classified according to albuminuria status: 47 (58.75%) had normoalbuminuria (≤30 mg/24h), 33 (41.25%) had microalbuminuria (30-300 mg/24h), and 10 healthy individuals served as controls. There were no statistically significant differences among the groups regarding age, sex, body mass index (BMI), blood pressure, smoking habits, or diabetes duration. The mean BMI was 26.64±3.56 kg/m^2^ in the normoalbuminuric group, 28.38±5.31 kg/m^2^ in the microalbuminuric group, and 25.73± 4.77 kg/m^2^ in the control group. As expected, albuminuria levels differed significantly between groups (p<0.001). The baseline demographic and clinical characteristics of the study population are summarised in Table 1.

**Table 1:** Baseline clinical characteristics of patients according to levels of urinary albumin (25). All data are expressed as means ± SD except smoking habits. DM - diabetes mellitus; BMI - body mass index; HbA1c - glycosylated hemoglobin A1C; GFR - glomerular filtration rate.

Characteristics of<br>the patients	All Patients<br>(n=80)	Normoalbuminuric <br>(n=47)	Microalbuminuric <br>(n=33)	Healthy<br>(n = 10)	p
	x̄±SD	x̄±SD	x̄±SD	x̄±SD	
Gender (M/F)	44/36	27/20	17/16	5/5	0.276
Age (Years)	59.85±8.871	60.49±8.73	58.94±9.13	54±10.59	0.014
Duration of DM (Years)	13.29±7.69	13.34±7.74	13.21±7.73	n/a	0.942
BMI (kg/m^2^)	27.36±4.42	26.64±3.56	28.38±5.31	25.73±4.77	0.325
Current smoker (%)	39 (48.8 %)	26 (55.3%)	13 (39.4%)	3 (30%)	
Systolic BP (mmHg)	134.60±14.08	133.47±12.87	136.21±15.71	122±17.02	0.411
Diastolic BP (mmHg)	81.56±7.53	80.96±7.42	82.42±7.72	75.5±10.39	0.389
Serum Creatinine (μmol/L)	75.38±15.04	76.64±15.97	73.58±13.64	73.6±7.6	0.360
GFR (mL/min /1.73 m^2^)	86.86±14.18	85.78±13.55	88.39±15.12	92.84±9.06	0.430
HbAIC (%)	7.59±1.34	7.25±1.15	8.07±1.45	4.93±0.3	0.074
Microalbuminuria	40.42±40.89	21.24±3.72	67.75±52.90	17.8±7.97	0.001

### Urinary biomarkers

Comparison of urinary biomarker concentrations between study groups using the Student's t-test revealed statistically significant differences for all analyzed markers. Morning urine values are presented.

Type IV collagen: Microalbuminuric patients had a mean concentration of 0.97 ng/mL, normoalbuminuric patients 0.55 ng/mL, and healthy controls 0.48 ng/mL (p<0.001).

Transferrin: Concentrations were 45.51 ng/mL in microalbuminuric patients, 14.83 ng/mL in normoalbuminuric patients, and 6.57 ng/mL in controls (p<0.001).

L-FABP: Values were 480.15 ng/mL for microalbuminuric patients, 228.08 ng/mL for normoalbuminuric patients, and 89.91 ng/mL for controls (p<0.001). Concentrations of urinary biomarkers in first-morning urine, stratified by albuminuria level, are shown in Table 2.

**Table 2:** The concentrations of urinary biomarkers according to albuminuria levels. Pearson's test, *r* - correlation coefficient; x̄ - mean; SD - standard deviation

Concentration of<br>biomarker<br>(ng/mL)	album. 30 mg/24h<br>(N=33)	album.<30 mg/24h<br>(N=47)	Healthy<br>(N = 10)	Pearson's <br>R	*P*
	x̄±SD	x̄±SD	x̄±SD	x̄±SD	
Colagen type IV<br>spot urine	0.97±0.17<br>1.00 (0.00-1.00)	0.55±0.29<br>0.53 (0.00-1.00)	0.48±0.23<br>0.57 (0.08-0.89)	0.384	<0.001
Transferrin - spot<br>urine	45.51± 28.13<br>35.60 (12.31-101.83)	14.83± 18.51<br>8.32 (1.28-98.98)	0.48±0.23<br>0.57 (0.08-0.89)	0.384	<0.001
L-FABP -<br>spot urine	480.15±297.95<br>331.38 (82.15-989.49	228.08±199.66<br>137.79 (1.53-655.81)	6.57±3.70<br>5.63 (2.22-1.81)	0.414	<0.001

Optimal cut-off values (p/n cut-off points) for DKD presence were determined, along with the corresponding maximum sensitivity and specificity for each biomarker. For type IV collagen, the maximum sensitivity of 72.27% and specificity of 83% were achieved at a urinary type IV collagen level of 0.630 ng/ml. The cut-off value for urinary transferrin was 21.62 ng/mL, yielding a maximum sensitivity of 78.8% and specificity of 78.7%. For L-FABP the lowest sensitivity and specificity were 69.7% and 70.2%, respectively, at a cut-off of 366.12 ng/mL. Table 3 presents the optimal cut-off values for the analyzed biomarkers.

**Table 3:** Optimal cut-off values for biomarkers. * Value at which maximum sensitivity and specificity were achieved

Biomarker	p/n cut off point*	Sensitivity (95% CI)	Specificity (95% CI)
Collagen type IV - spot urine<br>(ng/ml)	0.63	72.27	83.0
Transferrin - spot urine (ng/mL)	21.62	78.8	78.7
L-FABP - spot urine (ng/mL)	366.12	69.7	70.2

**Figure 1:**
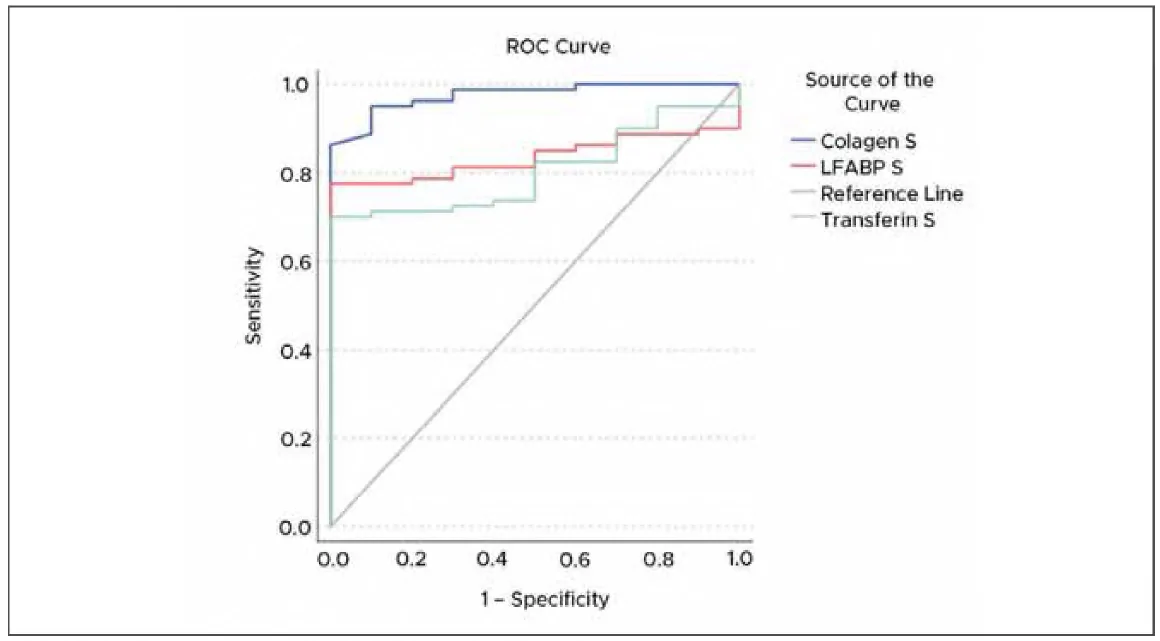
Accuracy of excretion of urinary biomarkers in spot urine.

To identify early diabetic kidney disease (DKD) before the onset of microalbuminuria, the diagnostic potential of all urinary biomarkers was assessed. Among the three biomarkers, type IV collagen in spot urine demonstrated the highest diagnostic potential, with an area under the ROC curve (AUC) of 90.4% and a 95% confidence interval (CI) of 84.2-96.5%. Urinary transferrin showed an AUC of 86.7% (95% CI: 79.0-94.3%), while L-FABP exhibited slightly lower diagnostic potential, with an AUC of 80.8% (95% CI: 71.0-90.1%) (Figure 1).

The diagnostic potential of urinary type IV collagen, transferrin, and L-FABP for the presence of diabetic kidney disease is presented in Table 4.

**Table 4:** Diagnostic potential of biomarkers. *Area under the curve ROC (DeLong-a); *Confidence interval

Biomarker	AUC ROC* with 95% CI*
Colagen type IV - spot urine (ng/mL)	90.4% (84.2%-96.5%)
Transferrin - spot urine (ng/mL)	86.7% (79.0%-94.3%)
L-FABP - spot urine (ng/mL)	80.8% (0.71%-90.1%)

In addition to evaluating urinary biomarkers for early detection of diabetic kidney disease, we also investigated the association between urinary biomarker concentrations and the presence of diabetic retinopathy (DR), another microvascular complication of diabetes mellitus. Based on ophthalmological examination, patients were divided into two groups: 24 patients (30%) with diabetic retinopathy and 56 patients (70%) without retinopathy. Among those with retinopathy, 17 patients (70.83%) had proliferative DR, while seven patients (29.17%) had non-proliferative DR.

No statistically significant differences in urinary type IV collagen and L-FABP concentrations were observed between patients with and without DR. However, urinary transferrin levels were significantly higher in patients with diabetic retinopathy. In the DR group, the mean urinary transferrin concentration was 81.38±75.52 ng/mL, compared with 38.12± 47.80 ng/mL in patients without retinopathy (p<0.03). These findings suggest that urinary transferrin correlates with the presence of DR in patients with type 2 diabetes.

In addition to urinary transferrin levels, diabetes duration also influenced the development of DR. Patients with DR had a mean disease duration of 19.96±7.35 years, whereas patients without DR had a mean duration of 10.42±5.87 years (p<0.001) (Table 5).

**Table 5:** Comparison of variables significant for the development of retinopathy.

Variable	Retinopathy (n = 26)	Without retinopathy (n=54)	P
	x̄±SD	x̄±SD	
Age (years)	62.66±8.07	58.64±8.98	0.540
BMI (kg/m^2^)	26.52±3.12	27.71±4.85	0.196
HbAIC (%)	7.49±1.36	7.62±1.34	0.690
Duration of DM (years)	19.96±7.35	10.42±5.87	<0.001
Microalbuminuria (mg/24h)	55.50±64.67	33.95±22.60	<0.001
Transferrin - spot urine (ng/mL)	81.38±75.52	38.12±47.80	<0.030
Colagen type IV - spot urine (ng/mL)	0.58±0.20	0.49±0.16	0.450
LFABP - spot urine (ng/mL)	715.75±505.25	728.62±665.43	0.300

**Figure 2:**
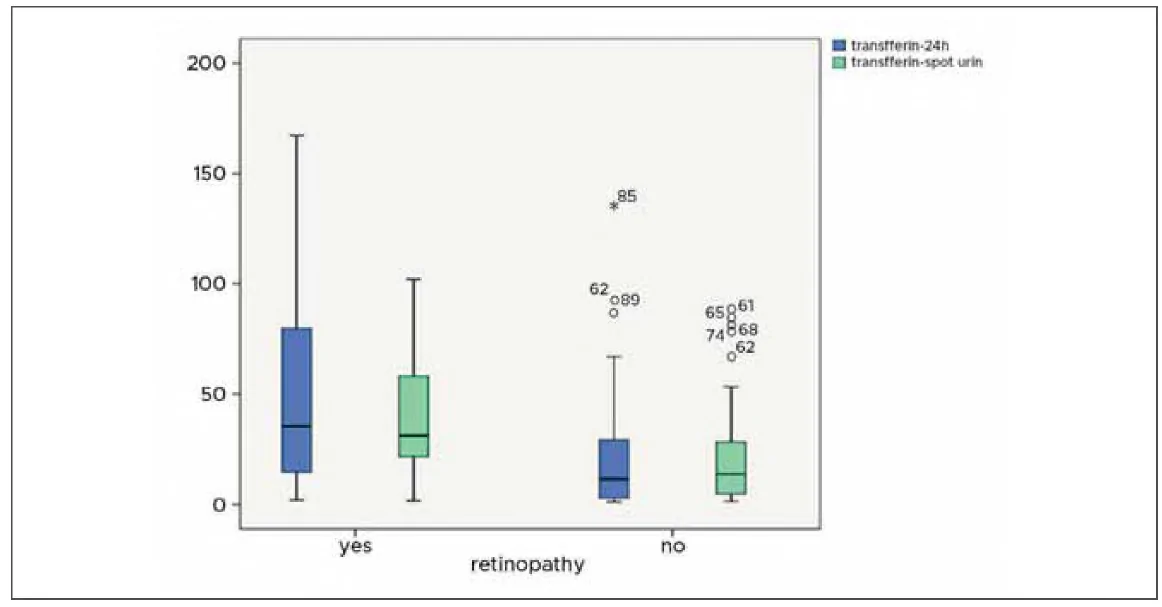
Comparison of urinary transferrin concentrations between patients with and without diabetic retinopathy.

The comparison between diabetic retinopathy and urinary transferrin is shown graphically, presenting values obtained from both spot and 24-hour urine samples, between which no statistically significant difference was observed. However, the previously mentioned difference in transferrin levels between individuals with and without diabetic retinopathy is evident. The results are presented in Figure 2.

## Discussion

Diabetes mellitus (DM) is a chronic systemic inflammatory disease frequently accompanied by microvascular complications, among which diabetic kidney disease (DKD) is the most prevalent. Although microalbuminuria remains the most widely used early indicator of DKD, growing evidence shows that many patients with reduced GFR do not exhibit albuminuria, highlighting the need for more sensitive and specific early biomarkers [25][26][27][28][29][30][31]. In this study, we evaluated urinary type IV collagen, transferrin, and L-FABP as markers of glomerular and tubular injury associated with DKD and diabetic retinopathy (DR).

In our cohort, 41.25% of patients had microalbuminuria. Urinary type IV collagen levels were significantly higher in this group (r=0.384; p<0.001), consistent with the multicenter study by Tomina et al. [32] and the findings of Hassan et al. [33], both of which support its role as an early marker of DKD.

Urinary transferrin also differed significantly among groups (r=0.456; p<0.001), with higher concentrations in patients with increasing levels of albuminuria. These results align with reviews and longitudinal studies by Cusmao et al. [19], Narita et al. [34], and Bangjian et al. [20], which collectively indicate that transferrinuria often precedes microalbuminuria and may predict DKD progression.

Urinary L-FABP levels were significantly associated with albuminuria (r=0.430; p<0.001), in agreement with previous findings that identify L-FABP as a sensitive marker of tubular injury and early DKD progression. Viswanathan et al. [35], Kamijo-Ikemori et al. [24], Thi et al. [36], and Panduru et al. [37], reported very similar results.

ROC curve analysis demonstrated good diagnostic performance for all three biomarkers: type IV collagen (AUC 90.4%), transferrin (AUC 86.7%), and L-FABP (AUC 80.8%). Transferrin showed the highest sensitivity (78.8%), while type IV collagen exhibited the highest specificity (83%), indicating their strong potential for early DKD detection.

Regarding diabetic retinopathy, only urinary transferrin showed a significant association with DR (p=0.003-0.014). This finding is consistent with the study by Zhang et al. [15], who also observed a higher prevalence of transferrinuria than microalbuminuria in patients with and without DR, suggesting that transferrin may serve as an indicator of generalised microvascular injury.

This pilot study demonstrated that urinary type IV collagen, transferrin, and L-FABP are elevated in patients with early DKD and exhibit meaningful diagnostic performance. These findings align with previous studies suggesting that early glomerular and tubular injury may be detectable before overt clinical nephropathy. Our results confirm urinary transferrin as a potential early biomarker of DKD and suggest a relationship with DR. However, the biological mechanisms linking glomerular filtration markers to retinal pathology remain incompletely understood.

## Conclusion

Urinary type IV collagen, transferrin, and L-FABP may serve as adjunctive biomarkers for early detection of DKD. Among them, type IV collagen demonstrated the strongest diagnostic performance, while urinary transferrin was also associated with diabetic retinopathy. As this is a pilot study, these findings require confirmation in larger multicenter cohorts.

## Dodatak

### Funding

There was no Funding Declaration in the manuscript.

### Ethical approval

The procedures used were in accordance with the ethical standards of the competent committee for experimentation on humans and with the Declaration of Helsinki of 1975, revised in 2000. The study was approved by the institutional ethics committee (Military Medical Academy).

### Authors' contribution

Brankica Terzić - conceptualization, methodology, software, validation, investigation, formal analysis, writing - original draft, writing - review & editing, visualization. Zoran Radojičić - conceptualization, methodology, software, validation, data curation, formal analysis. Predrag Đurić - conceptualization, methodology, investigation. Dušan Pualić - conceptua I ization, methodology, investigation. Mirko Resan - conceptualization, methodology, investigation, Mirjana Mijušković - conceptualization, methodology, software, validation, investigation, formal analysis, writing - original draft, writing - review & editing, visualization, resources, supervision.

### Conflict of interest statement

All the authors declare that they have no conflict of interest in this work.
